# Association of Apolipoprotein E Polymorphisms and Risks of Ischemic Stroke in Chinese Patients with Type 2 Diabetes Mellitus

**DOI:** 10.1155/2021/8816996

**Published:** 2021-01-03

**Authors:** Na Wang, Qian Liu, Hui Liu, Xiao Cong, Hui Yang, Yang Yu, Yongtong Cao, Liang Ma

**Affiliations:** ^1^Department of Transfusion Medicine, China-Japan Friendship Hospital, Beijing 100029, China; ^2^Clinical Laboratory, China-Japan Friendship Hospital, Beijing 100029, China

## Abstract

**Background:**

The apolipoprotein E (*APOE*) gene polymorphisms have been intensively studied in patients with type 2 diabetes mellitus (T2DM) and ischemic stroke (IS) in recent years. However, it is unclear whether *APOE* gene polymorphisms are correlated with increased risk for developing IS in T2DM patients. Thus, this study was designed to examine the association between *APOE* gene polymorphisms and risks of IS in Chinese patients with T2DM.

**Methods:**

This case-control study enrolled 243 subjects with T2DM as controls, and 210 subjects with T2DM complicated with IS as case patients. The genotypes were determined using real-time PCR while HbA1c and lipid levels were detected using commercially available kits.

**Results:**

The systolic blood pressure (SBP), diastolic blood pressure (DBP), and the proportion of patients with a history of hypertension were higher in the case patients than that in the controls. We confirmed that the *ε*2/*ε*3 genotype, as well as SBP and history of hypertension, was the independent risk factor for developing IS in T2DM patients.

**Conclusions:**

We conclude that the *ε*2/*ε*3 genotype might contribute to the increased risk for developing IS in Chinese patients with T2DM.

## 1. Introduction

China has the highest number of diabetes in the world. According to the International Diabetes Federation (IDF) report, there were 116 million Chinese diagnosed with diabetes in 2019, and the number will increase to 141 million by 2030 [1]. Ischemic stroke (IS) is a cerebrovascular complication of type 2 diabetes mellitus (T2DM) due to accelerated atherosclerosis and carotid artery disease development [2]. Both macrovascular and microvascular systems of the brain are severely affected in T2DM [3]. T2DM exacerbates ischemic brain injury and worsens functional outcome after stroke [4]. Patients with T2DM have a two- to four-fold increase in the risk of IS compared with the general population [5, 6]. Moreover, stroke patients with diabetes have a poor prognosis after stroke onset [7]. Thus, it is of great importance to investigate the risk factors of T2DM patients complicated with IS.

Mounting evidence indicates that the apolipoprotein E (*APOE*) gene located at chromosome 19q13.32 is a candidate gene in the development of T2DM and IS [8–10]. The protein encoded by the *APOE* gene consists of 299 amino acid residues and contains amphipathic *α*-helical lipid-binding structural domains which enable APOE to interact with members of the low-density lipoprotein receptor family. Through the interaction, APOE plays an essential role in lipid transportation in both plasma and brain [11]. The dysregulation of the *APOE* expression and genetic variance of the *APOE* influence the APOE functions and lead to the pathogenesis of nervous and cardiovascular diseases eventually [11–13]. The common gene variants, epsilon-2 (*ε*2), epsilon-3 (*ε*3), and epsilon-4 (*ε*4), are generated by the two single-nucleotide polymorphisms (SNPs) rs7412 (C/T) and rs429358 (C/T) in exon 4 of the *APOE* gene. The variants are different haplotypes of the *APOE* gene generated by the combination of the two SNPs at the *APOE* locus [14]. The most common allele isoform is *ε*3 with a frequency of 70-80%. The *ε*2 and *ε*4 have a frequency of 5-10% and 10-15%, respectively [15]. The 3 alleles of the *APOE* gene form 6 genotypes (*ε*2/*ε*2, *ε*2/*ε*3, *ε*2/*ε*4, *ε*3/*ε*3, *ε*3/*ε*4, *ε*4/*ε*4) [16]. The *ε*3/*ε*3 is the most common genotype with a frequency of approximately 67% [8]. The frequencies of *ε*3/*ε*4 and *ε*2/*ε*3 are lower than those of *ε*3/*ε*3, and *ε*2/*ε*2, *ε*4/*ε*4, and *ε*2/*ε*4 genotypes have the lowest frequencies [17].

Understanding the risk factors of T2DM patients complicated with IS may shed light into intervening the development of complications of T2DM. So far, studies on the association of *APOE* polymorphisms and IS risks in Chinese patients with T2DM are lacking. Thus, this study is designed to elucidate whether the *APOE* gene polymorphism is an essential determinant of Chinese patients with T2DM complicated with IS.

## 2. Materials and Methods

### 2.1. Subjects

A case-control study was carried out from July 2015 to July 2018. Informed consent was obtained from all enrolled subjects, and the design protocol was approved by the Ethics Committee of China-Japan Friendship Hospital. The data of the families, their medical history, and smoking habits of the patients was obtained by a questionnaire. Clinical examination including measurement of systolic blood pressure (SBP) and diastolic blood pressure (DBP) was applied. Anthropometric data (weight and height) were collected and used for BMI calculation. Hypertension was defined as blood pressure above 140/90 mmHg or taking antihypertensive drugs. Dyslipidemia was characterized by increased total cholesterol (TC ≥ 6.20 mmol/L), low-density lipoprotein cholesterol (LDL − C > 4.13 mmol/L), and triglyceride (TG > 2.25 mmol/L), or decreased high-density lipoprotein cholesterol (HDL − C < 1.03 mmol/L) [18]. These subjects were categorized into two groups: T2DM patients complicated with and without IS according to the criteria of the American Diabetes Association Classification 2010 [19].

1. *T2DM patients without IS (control group)*. This group consists of 243 subjects fulfilling the T2DM diagnostic criteria or under diabetes medication (oral and/or insulin) with no history or signs of any IS.
2. *T2DM patients complicated with IS (IS group)*. This group consists of 210 subjects diagnosed to have T2DM or under diabetes medication and complicated with IS. All the subjects in this group were examined by a qualified neurologist. The diagnosis of IS was confirmed by clinical symptoms or signs, laboratory results, and computed tomography (CT) or magnetic resonance imaging (MRI). Exclusion criteria included cardiac diseases, renal diseases, hepatic diseases, endocrine diseases, metabolic disorders, autoimmune diseases, skeletal disorders, and cancerous diseases.

### 2.2. DNA Isolation and APOE Genotyping

Blood samples were collected in vacuum tubes and stored at -20°C until processed. DNA was extracted from each blood sample using the genomic DNA purification kit (Xi'an Tianlong Science and Technology Co., Ltd., Xi'an, Shaanxi, China). The concentration of DNA was quantified using a NanoDrop 1000 spectrophotometer (Thermo Fisher Scientific, Waltham, MA, USA). Three *APOE* alleles (*ε*2, *ε*3, and *ε*4) were detected by an *APOE* Genotyping Kit (Sinochips Bioscience Co., Ltd., Zhuhai, Guangdong, China). Polymorphic alleles were identified by the fluorescence intensity of the hybridization sites.

### 2.3. Biochemical Analysis

Plasma levels of fasting TG, TC, LDL-C, and HDL-C were measured using the AU5800 automated biochemical analyzer (Beckman Coulter, Brea, CA, USA). HbA1c was detected using the D-10 Hemoglobin Testing System (Bio-Rad, Hercules, CA, USA).

### 2.4. Statistical Analysis

The data were statistically analyzed using SPSS version 24.0 software (IBM, Chicago, IL, USA). Quantitative data were expressed as mean values ± standard deviation (SD). Normally distributed data were compared using Student's *t*-test. The significance of differences in the proportion of patients with a history of hypertension between the two groups was tested by the chi-square test (*χ*^2^). The differences in the distribution frequencies of genotype and allele between the two groups, and the deviations from Hardy-Weinberg equilibrium were tested by the chi-square test. The extremely rare genotype groups—*ε*2/*ε*2, *ε*2/*ε*4, and *ε*4/*ε*4—were excluded from the genotype and allele analyses. Univariable logistic regression analysis was used to test the association between IS and *APOE* gene polymorphisms, and the analysis results were presented as unadjusted odds ratios (OR) with confidence intervals (95% CI). Multivariate logistic regression was used to determine the risk factors for developing IS in T2DM patients with adjustment for potential covariates: age, gender, BMI, blood pressure, duration of T2DM, smoking, HbA1c level, and plasma lipids, and the results were presented as adjusted ORs. *P* < 0.05 was considered to be statistically significant.

## 3. Results

### 3.1. General Characteristics and Biochemical Variables of the Patients

210 T2DM patients complicated with IS and 243 age- and sex-matched controls from the same demographic area were included in this study.  Table 1 presents the general characteristics and biochemical variables of the patients in the control and the IS groups. No statistically significant differences were observed between the groups in age, gender, BMI, smoking, diabetes duration, HbA1c level, and plasma lipid level (*P* > 0.05). However, SBP, DBP, and the proportion of patients with a history of hypertension were higher in the IS group than in the control group (*P* < 0.05).

### 3.2. APOE Genotype and Allele Distribution Frequencies in the IS and the Control Groups

The *APOE* genotype distribution in the IS and the control groups were in Hardy-Weinberg equilibrium (*P* > 0.05). Distribution frequencies of the 6 genotypes and 3 alleles of *APOE* in the IS and the control groups are summarized in Table 2.

### 3.3. Association of APOE Gene Polymorphisms and IS

The univariate analysis was used to evaluate the association from the perspective of the genotype and allele frequencies of *APOE* gene polymorphisms. The genotype of *ε*2/*ε*3 increased the risk of IS in T2DM patients, with unadjusted OR 1.901 (95% CI 1.117-3.297,*P* = 0.0179), while the genotype of *ε*3/*ε*4 showed no association with IS in T2DM patients, with unadjusted OR 1.629 (95% CI 0.943-2.756,*P* = 0.0694) (Table 3). The results displayed no significant difference in *APOE* gene allele frequencies between the two groups (Table 3). After being adjusted for age, gender, BMI, SBP, DBP, history of hypertension, smoking habits, diabetes duration, HbA1c, TG, TC, LDL-C, HDL-C, and history of dyslipidemia using multivariable binary logistic regression analysis as shown in Table 4, the *ε*2/*ε*3 genotype was an independent risk factor for developing IS in T2DM patients, with adjusted OR 2.225 (95% CI 1.244-3.980, *P* = 0.007). However, the genotype of *ε*3/*ε*4 was not found to be an independent risk factor for developing IS in T2DM patients, with adjusted OR 1.727 (95% CI 0.978-3.049, *P* = 0.060) (Table 4). SBP (95% CI 1.004-1.031, *P* = 0.013) and history of hypertension (95% CI 1.089-3.045, *P* = 0.022) were also independent risk factors for developing IS in T2DM patients (Table 4).

## 4. Discussion

Many studies showed that patients suffering from T2DM are at a higher risk for IS than individuals without T2DM [4, 20]. Thus, it is essential to explore the predisposing risk factors for IS among T2DM patients. We carried out a case-control study to investigate the association of the *APOE* gene polymorphisms and risks of IS in Chinese patients with T2DM. We observed that SBP, DBP, and the proportion of patients with a history of hypertension were higher in the IS group than in the control group. We also found that the *ε*2/*ε*3 genotype was an independent risk factor for developing IS in T2DM patients. SBP and history of hypertension were also independent risk factors for developing IS in T2DM patients after adjusting for age, gender, BMI, SBP, DBP, history of hypertension, smoking habits, diabetes duration, HbA1c, TG, TC, LDL-C, HDL-C, and history of dyslipidemia.

A prospective population-based study, with approximately 20-year follow-up, evaluated the effect of T2DM on cardiovascular disease in 13105 samples. The study revealed an increased relative risk for developing stroke of 1.5 to 6.5 fold in T2DM individuals [20]. The increased risk is seen even after early diagnosis. It is reported that the risk of stroke in newly treated patients with T2DM is 9.1% within the first 5 years. The stroke rate is double that of the general population [21]. Many studies have indicated that T2DM patients have residual neurological deficits and a worse functional outcome, along with worse long-term mortality [7, 22]. Therefore, patients and physicians need to aggressively control cardiovascular risk factors soon after T2DM diagnosis. The identification of susceptibility genes and other risk factors would be helpful for the management of IS in T2DM patients.

*APOE* gene is one of the most widely studied candidate genes of T2DM. In a case-control study recruiting of 451 Thais, it has been demonstrated that *ε*4 allele containing genotypes were the predictors of T2DM [23]. A meta-analysis of 30 studies including 5423 case patients and 8197 controls suggests that *ε*2 allele is associated with increased risk of T2DM [8]. Besides, the relation between *APOE* gene polymorphisms and IS has been intensively investigated in many studies. Most findings suggest that *ε*4 allele increases the odds of IS [24–26]. However, little is known about the link between *APOE* gene polymorphisms and developing IS in T2DM patients. So far, there is only one study conducted on the issue. The study displayed that *APOE* gene polymorphisms were not linked with IS in T2DM patients [27]. However, we found that T2DM patients who carried the *ε*2/*ε*3 genotype were at 1.90-fold increased risk to develop IS in the present study. After adjustment for other established risk factors, *ε*2/*ε*3 was an independent risk factor for developing IS in T2DM patients. The discrepancies of the findings may be due to ethnic differences, sample sizes, genotyping method, and other risk factors. *APOE* gene polymorphisms affect plasma lipid concentration and may be responsible for the development of IS onset in T2DM patients. Further investigation is needed to study the *APOE* gene polymorphisms and lipid metabolism in T2DM patients complicated with IS.

Consistent with our results, many studies have shown that T2DM patients with hypertension tend to have a higher risk of getting IS [2, 28, 29]. We found that SBP, DBP, and the proportion of patients with a history of hypertension were higher in the IS group compared with that in the control group. Furthermore, SBP and history of hypertension were independent risk factors for developing IS in T2DM patients after adjusting for many related factors. So, proper blood pressure management is a core issue for mitigating the development of IS in T2DM patients.

There are some limitations in our study which require further investigation. Firstly, the replicability of the data needs to be verified in a larger sample size involving different ethnic groups. Secondly, our study is inadequate in proving the causal relationship but merely an association research due to the study design of case-control. However, our findings indeed contribute to risk prediction for IS in T2DM patients. The genotype *ε*2/*ε*3 of *APOE* is possibly a genetic predisposition factor for IS in T2DM patients. Furthermore, it is of great significance to control the blood pressure for T2DM patients to avoid IS onset.

## 5. Conclusions

Our study indicates that the *APOE* gene polymorphisms are associated with the development of IS in T2DM patients. We also identify *ε*2/*ε*3 genotype, SBP, and history of hypertension as independent risk factors in the development of IS in T2DM patients.

This study provides data that may help to improve the ability to identify diabetic individuals at increased risk for IS and improve the clinical management of patients with T2DM.

## Figures and Tables

**Table 1:** Demographic, clinical, and biochemical data of the IS and the control groups.

	IS (*n* = 210)	Control (*n* = 243)	*P* value
Age (years)	65.41 ± 8.20	64.58 ± 10.21	0.338
Gender (male %)	47.62	52.26	0.324
BMI (kg/mm^2^)	25.29 ± 3.98	25.75 ± 3.19	0.175
SBP (mmHg)	142.67 ± 19.41	133.98 ± 18.63	<0.001
DBP (mmHg)	81.64 ± 10.17	78.17 ± 10.67	<0.001
Hypertension (%)	84.28	70.20	<0.001
Smokers (%)	18.57	17.70	0.809
Diabetes duration (years)	14.44 ± 6.09	15.10 ± 8.32	0.332
IS duration (years)	3.65 ± 2.10	—	—
HbA1c (%)	7.66 ± 1.72	7.96 ± 1.88	0.084
HbA1c (mmol/mol)	60.25 ± 18.81	63.45 ± 20.59	0.084
TG (mmol/L)	1.88 ± 1.62	2.00 ± 1.46	0.433
TC (mmol/L)	4.20 ± 1.24	4.24 ± 1.14	0.697
LDL-C (mmol/L)	2.48 ± 0.95	2.52 ± 0.99	0.666
HDL-C (mmol/L)	1.05 ± 0.33	1.04 ± 0.31	0.880
Dyslipidemia (%)	64.29	64.20	0.984

**Table 2:** Distribution frequencies of *APOE* genotype and allele in the IS and the control groups.

	*APOE* genotypes (%)						*APOE* alleles (%)		
	*ε*2/*ε*2	*ε*2/*ε*3	*ε*2/*ε*4	*ε*3/*ε*3	*ε*3/*ε*4	*ε*4/*ε*4	*ε*2	*ε*3	*ε*4
IS	2 (0.95)	39 (18.57)	4 (1.90)	126 (60.00)	37 (17.62)	2 (0.95)	47 (11.19)	328 (78.10)	45 (10.71)
Control	45 (1.65)	28 (11.52)	7 (2.88)	172 (70.78)	31 (12.76)	1 (0.41)	43 (8.85)	403 (82.92)	40 (8.23)

**Table 3:** Associations of *APOE* gene polymorphisms and the risk of IS in T2DM patients compared to that in case patients, represented as unadjusted OR.

	Unadjusted OR (95% CI)	*P* value
Genotype		
*ε*2/*ε*3 *vs. ε*3/*ε*3	1.901 (1.117-3.297)	0.0179
*ε*2/*ε*3 *vs. ε*3/*ε*4	1.167 (0.602-2.281)	0.657
*ε*3/*ε*4 *vs. ε*3/*ε*3	1.629 (0.943-2.756)	0.0694
Allele		
*ε*2 *vs. ε*3	1.343 (0.876-2.073)	0.186
*ε*2 *vs. ε*4	0.972 (0.534-1.766)	0.924
*ε*4 *vs. ε*3	1.382 (0.882-2.190)	0.157

The extremely rare genotypes—*ε*2/*ε*2, *ε*2/*ε*4, and *ε*4/*ε*4 subjects—were excluded from statistical analysis.

**Table 4:** Associations of *APOE* gene polymorphisms and the risk of IS in T2DM patients compared to that in case patients, represented as adjusted OR.

	Adjusted OR (95% CI)	*P* value
*ε*3/*ε*3 (referee)	—	0.010
*ε*2/*ε*3	2.225 (1.244-3.980)	0.007
*ε*3/*ε*4	1.727 (0.978-3.049)	0.060
Age	1.009 (0.986-1.033)	0.452
Gender	0.880 (0.570-1.358)	0.563
BMI (kg/mm^2^)	0.958 (0.901-1.018)	0.166
SBP (mmHg)	1.017 (1.004-1.031)	0.013
DBP (mmHg)	1.017 (0.993-1.042)	0.168
Hypertension (%)	1.821 (1.089-3.045)	0.022
Smokers (%)	1.172 (0.672-2.042)	0.576
Diabetes duration	0.981 (0.954-1.009)	0.186
HbA1c	0.896 (0.798-1.006)	0.063
TG	0.925 (0.772-1.109)	0.398
TC	1.032 (0.580-1.835)	0.916
LDL-C	0.921 (0.496-1.710)	0.793
HDL-C	0.988 (0.387-2.517)	0.979
Dyslipidemia	1.458 (0.841-2.529)	0.179

## Data Availability

The data used to support the findings of this study are available from the corresponding authors upon request.
